# Robot-assisted Tongue Base Resection ensures favorable therapeutic outcome to Obstructive Sleep Apnea patients with Lingual tonsil hypertrophy

**DOI:** 10.1038/s41598-018-36800-7

**Published:** 2019-01-24

**Authors:** Sung-Woo Cho, Seung-No Hong, Doo Hee Han, Tae-Bin Won, Dong-Young Kim, Hyun Jik Kim

**Affiliations:** 1 Department of Otorhinolaryngology, Seoul National University College of Medicine, Seoul National University Hospital, Seoul, Korea; 2 Department of Otorhinolaryngology, Seoul National University College of Medicine, Seoul National University Bundang Hospital, Seongnam, Korea; 3 Department of Otorhinolaryngology, Seoul National University College of Medicine, Borame Medical Center, Seoul, Korea

## Abstract

Tongue base (TB) narrowing is recognized as a significant site of upper airway collapse during sleep in obstructive sleep apnea (OSA) patients and robot technology is expected to have promising clinical utility in OSA patients with TB narrowing. The purpose of our study is to demonstrate the better therapeutic conditions and favorable indications of robot-assisted TB resection (TBR) in OSA. We performed robot-assisted TBR combined with nasal and palatal surgery in 16 OSA patients with any of the following characteristics: severe TB narrowing (over grade II) and moderate or severe OSA. The preoperative median AHI was 48.8/hr and the median lowest SaO_2_ was 82.0%. The median AHI decreased to 18.7/hr and ten patients (62.5%) were included in the responder group following robot-assisted TBR combined with nasal and palatal surgery. The lowest SaO_2_ improved to 90.5% and the posterior airway space (PAS) was significantly increased following robot-assisted TBR. Cephalometric results showed that wider PAS were observed in responders compared to non-responders prior to robot-assisted TBR. Interestingly, there was greater improvement in the objective parameters including PAS in the OSA patients with lingual tonsilar hypertrophy than they were in those without and all patients with lingual tonsillar hypertrophy (n = 6) responded to robot-assisted TBR. Robot-assisted TBR exhibited minimal morbidity and postoperative complications in OSA patients. Robot-assisted TBR can be considered a promising and innovative surgical option to reduce TB volume and improve sleep parameters in OSA patients with TB narrowing. OSA patient with TB narrowing due to lingual tonsil hypertrophy shows greater therapeutic outcome and lingual tonsil hypertrophy appears to be most favorable surgical indications of robot-assisted TBR.

## Introduction

Obstructive sleep apnea (OSA) is a common sleep disorder that is characterized by repeated episodes of partial or complete upper airway collapse during sleep^1^. This airway collapse results in reduction or cessation of airflow, which increases the airway resistance and contributes to the underlying pathogenesis of OSA showing loud snoring and repeated apneic events during sleep. If not treated properly, OSA leads to intermittent hypoxia, increased sympathetic overdrive, and excessive oxidative stress; therefore, it may cause fatal systemic complications to OSA patients^2–5^. Several different therapeutic options, including both medical and surgical interventions, have been proposed to reduce upper airway narrowing and airway resistance in OSA patients.

OSA is characterized a fixed or dynamic upper airway obstruction and this obstruction is either caused by anatomical factors or abnormal upper airway motor tone. For instance, the upper airway may be obstructed by the collapse of single or multiple structures, such as the soft palate, uvula, palatine tonsils, lateral pharyngeal walls, and tongue base (TB)^6,7^. The various treatment modalities for OSA attempt to widen the upper airway and reduce airway collapsibility. Some of the options include behavioral modifications, continuous positive airway pressure (CPAP), surgery, and oral appliances^8^. The surgical treatments for OSA primarily aim to remove the redundant oropharyngeal tissues that block the upper airway. Removing these tissues enhances the stability of the pharyngeal airway and maintains the pharyngeal lumen during sleep to minimize excessive upper airway collapse. Many studies have demonstrated clinical benefits of sleep surgeries, including relief from both subjective symptoms and life-threatening conditions^9–11^. Diverse surgical techniques have been introduced to correct abnormal upper airway narrowing and the addition of new surgical options can potentially improve the therapeutic outcomes^11,12^.

TB narrowing can be a predominant anatomic factor inducing airway narrowing or collapse in OSA patients and has been recognized to contribute independently to the pathogenesis of OSA by aggravation of upper airway resistance, combined with partial or complete obstruction at the level of palate^13,14^. In addition, excessive narrowing and complete collapse at the level of TB is more frequently observed in severe OSA patients and TB narrowing is regarded as a significant site of obstruction in most OSA patients who fail surgical treatment^14^. Our previous clinical data showed incomplete correction of anatomic structures causing TB narrowing in moderate or severe OSA is closely related with a higher recurrence of apneic events and OSA surgical treatment failure^15^. Therefore, there is need for more complete control of TB narrowing in order to improve the therapeutic outcomes of OSA surgery and we need to drive more interest in improving surgical techniques for reduction of tongue base volume. According to recent published data, there are also several minimally invasive transoral techniques that have been introduced for volume reduction of TB in OSA patients. Some of these new techniques include radiofrequency ablation, laser-assisted oropharyngeal surgery, and coblation endoscopic lingual lightening^16–18^ and robot technology has been also successfully applied in correction of TB narrowing of OSA patients.

Robot-assisted tongue base resection (TBR) permits multi-planar tissue transaction at any angle for more effective resection of lymphoid tissue in TB. Several studies have suggested improved surgical access to TB, with higher success rates of TB volume reduction in OSA surgery. Robot-assisted TBR has been successfully applied in OSA patients worldwide, demonstrating its safety, efficacy, and tolerability^19^. We also assume that robot-assisted TBR is the most innovative technique for correcting TB narrowing, preventing collapse, and reducing the number of apneic events in OSA patients. However, there remains a critical need to determine the favorable surgical indications for OSA patients who underwent robot-assisted TBR for TB narrowing. In addition, careful patient selection is crucial to maximize the effectiveness of robot-assisted TBR in OSA patients.

In this study, we sought to determine the therapeutic outcomes of robot-assisted TBR combined with nasal and palatal surgeries in patients with moderate or severe OSA. We also investigated the favorable surgical indications for robot-assisted TBR using sleep parameters, drug-induced sleep endoscopic (DISE) findings, or cephalographic findings.

## Methods

### Ethics statement

Sixteen adult subjects who had been diagnosed with OSA at Seoul National University Hospital (Seoul, Korea) from January 2016 and July 2017 participated in the study. All subjects participated in the study voluntarily and the medical records of the participants were reviewed retrospectively. Written informed consent was obtained from each participant and the study complied with the Declaration of Helsinki. The institutional board of Seoul National University Hospital approved this study.

### Patients and Study Design

**Figure 1:**
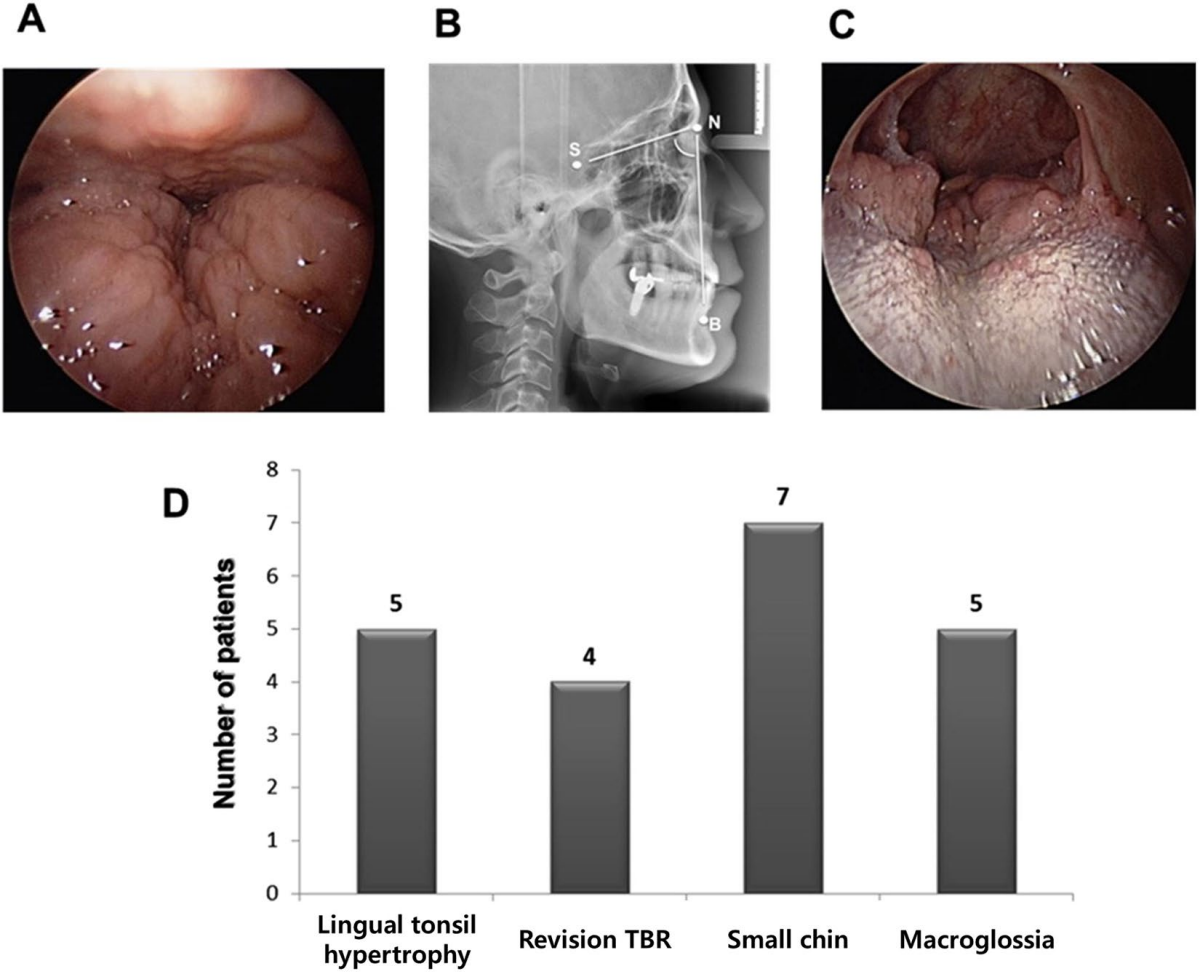
The surgical indications for robot-assisted TBR. (**A**) 30° endoscopic findings of lingual tonsil hypertrophy. (**B**) Cephalographic measurement for retrognathia (S: sella, N: nasion, B: infradental) (**C**) 30° endoscopic findings of remained TB lymphoid tissue in OSA patients with previous TB surgery. (**D**) Inclusion criteria for robot-assisted TBR in the present study. Bars represent the number of subjects.

**Figure 2:**
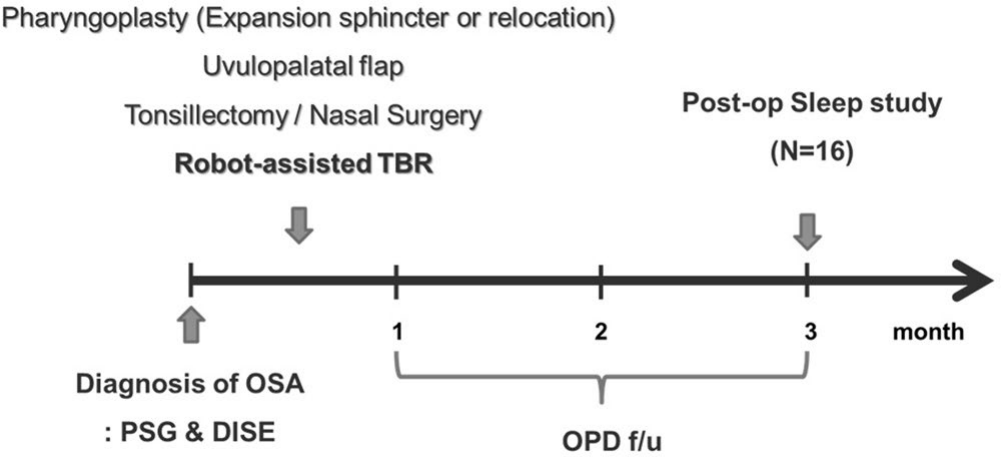
Schematic of the study design and clinical evaluation. (TBR: tongue base resection, PSG: polysomnogram, DISE: drug-induced sleep endoscopy).

Sixteen OSA subjects who were diagnosed with TB narrowing on DISE were included. All of the patients either could not tolerate or refused CPAP. All subjects underwent palatal surgery (expansion sphincter pharyngoplasty or relocation pharyngoplasty), tonsillectomy, uvulopalatal (UP) flap, septoturbinoplasty, and robot-assisted TBR to improve their sleep-related symptoms and abnormal sleep parameters. The inclusion criteria for robot-assisted TBR were as follows: (1) apnea-hypopnea index (AHI) >15 events/hr on polysomnography (PSG) and (2) TB narrowing above DISE grade II (more than 75% narrowing). Abnormalities of the TB were categorized as follows; huge lingual tonsils (Fig. 1A); retrognathia <78 degrees of the SNB angle (S: sella, N: nasion, B: infradental, Fig. 1B); TB narrowing despite of previous TB surgery (Fig. 1C) and severe narrowed oropharynx with macroglossia (Friedman palatal position IV) (Fig. 1D). Their medical records, including the results of pre- or postoperative PSG, were reviewed retrospectively. Valid sleep time, AHI (events/hr), and lowest O_2_ saturation were observed before and three months after the surgery (Fig. 2). A successful procedure was defined as a 50% reduction in AHI and an AHI less than 20, as described by Sher^20^. The following parameters were recorded at discharge from the hospital and during follow-up visits: duration of hospital stay, early or late complications, and subjective parameters such as the Epworth Sleeping Scale (ESS). To evaluate changes in the pharyngeal airway at the retroglossal levels, the posterior airway space (PAS) was evaluated. The PAS values were measured both preoperatively and three months postoperatively using cephalography.

### Surgical Technique for Robot-assisted TBR

**Figure 3:**
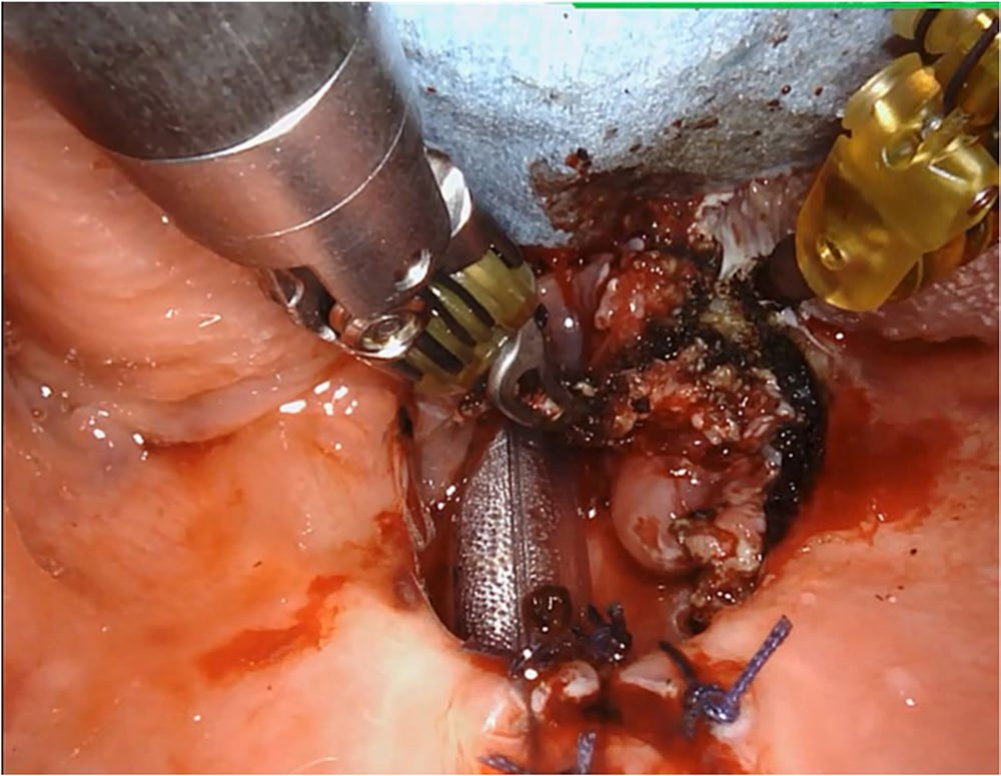
The surgical field of the robot-assisted TBR under da Vinci Xi.

The OSA subjects were selected for robot-assisted TBR based on the presence of TB narrowing, as determined by a complete preoperative upper airway examination during DISE using the VOTE classification. All subjects were scheduled to receive septoturbinoplasty, in addition to tonsillectomy and palatal surgery to improve the nasal and oropharyngeal airflow. Tracheostomy was not performed in all subjects who underwent robot-assisted TBR. Robot-assisted TBR was performed under general anesthesia with the patient in the Rose position and nasotracheal intubation was considered the first option whenever possible. In this situation, the nasotracheal tube was posterior to the center of the surgical field and was easily manipulated by the head assistant as needed. After ensuring feasibility of the TBR by way of TB inspection with a 30° degree endoscope, the Mclvor mouth gag retractor **(**Sklar Instruments, PA, USA) was placed in the airway for excellent TB exposure. A wider blade was preferred if possible but we did not use tooth- or soft tissue-protective devices, as these may reduce the baseline narrow mouth opening commonly observed in OSA subjects. The robot was arranged on the patient’s right side. After insertion of the Mclvor retractor, the robotic arms of the da Vinci system (Intuitive Surgical, Inc., Sunnyvale, CA, USA) were placed in the oral cavity. Visualization was achieved using a 30° high magnification, 3-dimensional endoscope. The surgery began with visualization of the epiglottis for orientation. The TBR procedure was then performed using a 8-mm Maryland dissector and 5-mm spatula tip cautery. First, the medial and paramedical portions of TB were addressed, followed by the lateral parts and this method allowed for identification and preservation of the noble structures. Under 30° visualization, the da Vinci system was employed to perform en bloc resection of huge lingual tonsils or hypertrophic TB tissue. The da Vinci system works from the circumvallate papillae to the midline in the vallecular and to tonsillar fossa laterally (Fig. 3). The resected tissue was predominantly lymphoid tissue, including a lingual tonsil. We formed limited piecemeal resection, which focused on redundant mucosal soft tissue around the lateral wall of the pharynx. Care was taken to avoid injury to the branches of the glossopharyngeal nerve and dorsal lingual artery. At the end of the procedure, hemostasis was obtained using the coagulation mode of the coblator (Evac70, Smith & Nephew, London, UK). All of the subjects were discharged two days after robot-assisted TBR. The last follow-up visit was performed three months postoperatively.

### Statistical Analysis

Statistical calculations were performed using SPSS 19.0 (IBM Corp., Armonk, NY). Postsurgical changes in AHI, lowest oxygen saturation, ESS value, VAS for snoring or apnea, and the PAS at the retroglossal space were analyzed using paired t-test or the Wilcoxon signed rank test and Mann-Whitney U test for subgroup analysis. Descriptive data are presented as mean ± standard deviations or median with interquatile ranges (IQRs). *P*-values < 0.05 were considered statistically significant.

## Results

### Therapeutic outcomes of robot-assisted TBR combined with nasal and surgeries

We recruited 16 subjects previously diagnosed with OSA and performed robot-assisted TBR combined with septoturbinoplasty and palatal surgery to resolve upper airway narrowing (including the TB narrowing). Twelve patients were men, and four were women. The mean age was 44.6 years (range, 17–70 years), and the mean BMI was 26.2 kg/m^2^ (range, 18.2–33.1 kg/m^2^). The severity of OSA was based on AHI; 6 patients demonstrated moderate OSA, while 10 were diagnosed with severe OSA. We did not recommend robot-assisted TBR to patients with mild OSA, even if they demonstrated more than grade 2 retroglossal area collapse on DISE findings. The mean robotic set-up and console time were 22.3 ± 4.5 and 38.6 ± 5.2 min, respectively. The mean duration for the robotic-assisted TBR procedure was 42.6 ± 8.5 min.

**Figure 4:**
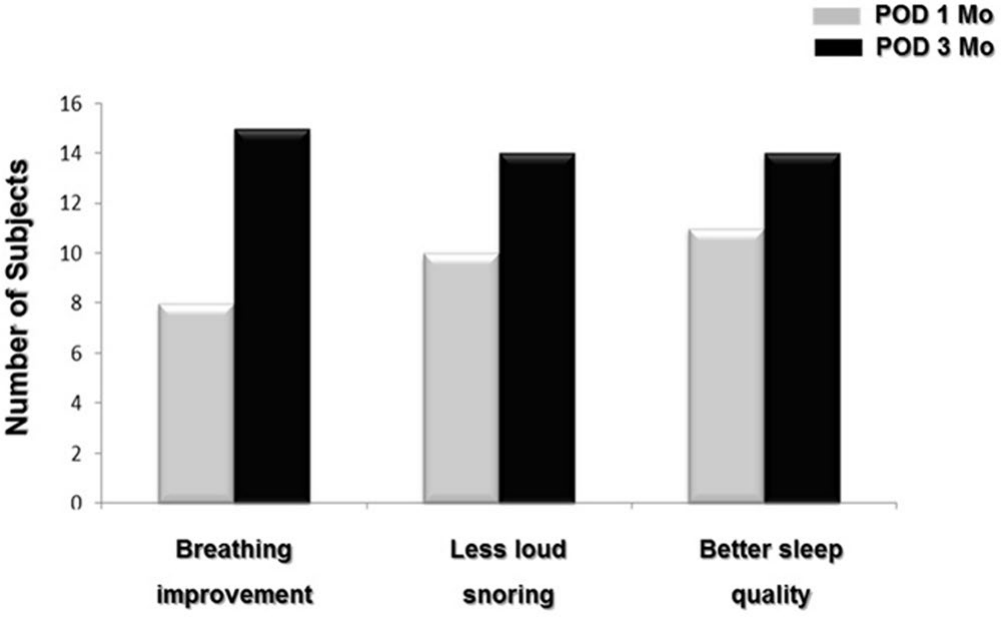
Changes in subjective symptoms of OSA patients following robot-assisted TBR. Improvement in subjective symptoms such as breathing, snoring volume, and sleep quality were determined using the visual analogue scale questionnaire.

The therapeutic outcome was determined using AHI after robot-assisted TBR combined with nasal and palatal surgeries. All of the subjects underwent PSG three months after surgery. The median AHI for the entire group was (IQR: 46.8) prior to multilevel surgeries. Three months after surgeries, the median AHI was 18.7/hr (IQR: 21.4) and it was significantly reduced compared to preoperative AHI (p = 0.006). Based on the arbitrary threshold of a 50% reduction in AHI and an AHI less than 20, the number of responders was 10, for a surgical success rate of 62.5% in OSA patients with TB narrowing. The postoperative PSG results also demonstrated that the lowest O_2_ saturation improved significantly from 82.0% (IQR: 21.6) to 90.5% (IQR: 10.9) (p = 0.013). In addition, postoperative valid sleep time increased from 313.5 ± 27.2 to 341.9 ± 31.5 min (p = 0.048) (Table 1). With regard to daytime sleepiness, the mean ESS value improved from 17.6 ± 4.8 prior to sleep surgery to 7.4 ± 5.8 one month postoperatively and 7.1 ± 3.2 at three months (p = 0.004). There were significant improvements in the subjective symptoms (including breathing during sleep, snoring, and subjective sleep quality) in all 16 subjects up to three months after robot-assisted TBR combined with nasal and palatal surgeries (Fig. 4).

**Table 1:** Changes in sleep parameters and PAS of OSA patients following robot-assisted TBR.

	Pre-op value	Post-op value	*p*-value
AHI	48.8/hr (IQR: 46.8)	18.7/hr (IQR: 21.4)	0.006
Lowest O_2_ saturation	82.0% (IQR: 21.6)	90.5% (IQR: 10.9)	0.013
Total sleep time	313.5 ± 27.2 min	341.9 ± 31.5 min	0.048
Epworth Sleepiness Scale	17.6 ± 4.8	7.1 ± 3.2	0.004
PAS	10.6 mm (IQR: 6.8)	18.2 mm (IQR: 4.6)	0.003

PAS: Posterior Airway Space.

We also measured changes in the distance of the PAS for a more detailed analysis of the clinical effect of robot-assisted TBR at the level of retroglossal area. The cephalographic data showed that the median PAS at the retroglossal area levels of the 16 subjects who underwent robot-assisted TBR significantly increased from 10.6 mm (IQR: 6.8) to 18.2 mm (IQR: 4.6) (p = 0.003) (Table 1). The mean admission duration was 4.0 ± 0.4 days. All subjects were transferred to general ward and tracheostomy was not performed in any of the subjects following robot-assisted TBR combined with nasal and palatal surgeries.

**Figure 5:**
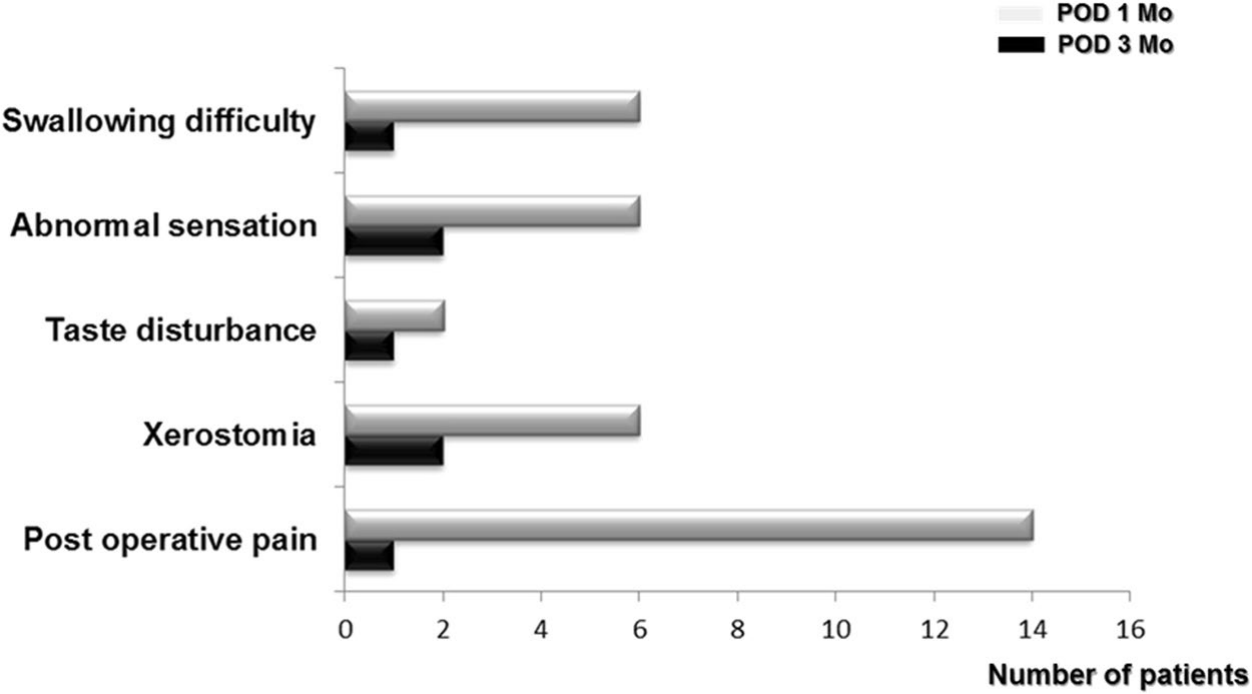
Subjective complaints and side effects one month and three months following robot-assisted TBR. Bars represent the number of subjects.

We next investigated the complications or side effects following robot-assisted TBR combined with nasal and palatal surgeries. Some of the potential complications include swallowing difficulty, abnormal sensation, taste loss, xerostomia, and postoperative pain for up to three months after surgery. Postoperative pain was most common symptom in OSA subjects having robot-assisted TBR and in total, 14 patients complained of pain at one month after robot-assisted TBR. However, only one patient experienced oropharyngeal pain until three months after robot-assisted TBR. In addition, six patients experienced an abnormal sensation around the oropharynx one month after surgery. Only two of these patients continued to have these symptoms at three months postoperatively. Some of the patients complained of swallowing difficulties, xerostomia, and taste loss after robot-assisted TBR; however, all of these complaints subsided by the third postoperative month (Fig. 5). We did not observe any post-operative bleeding at the TB.

### Favorable surgical indications for robot-assisted TBR

In particular, we recommend robot-assisted TBR for patients with moderate or severe OSA with the following features: TB narrowing above DISE grade II (more than 75% narrowing). The number of OSA subjects with TB abnormalities were as follows; lingual tonsil hypertrophy (n = 6), retroagnathia (n = 6), and macroglossia (Friedman palatal position IV) and orotopharyngeal narrowing in the absence of lingual tonsil hypertrophy or retroagnathia (n = 4). Four patients had remaining TB narrowing despite of previous TB surgery.

As described above, 10 patients could be classified into responders following robot-assisted TBR combined with nasal and palatal surgeries. Interestingly, all six subjects with huge lingual tonsils (100%) were in the responder group. Three subjects (42.8%) with retrognathia were also responders after robot-assisted TBR as a part of multilevel surgeries and robot-assisted TBR provided successful outcome in two patients (50%) who had TB surgery before. There was no significant difference in preoperative AHI among responders and non-responders (p = 0.313). The cephalographic results revealed that median preoperative PAS was 8.2 mm (IQR: 8.0) in the responders and 13.8 mm (IQR: 6.4) in non-responders, and responders had significantly narrower preoperative PAS compared to non-responders prior to robot-assisted TBR (p = 0.016). The median difference of preoperative and postoperative PAS was 9.5 (IQR: 10.5) in the responders and 3.5 (IQR: 4.8) in non-responders. The change of PAS was greater in responders compared to non-responders following robot-assisted TBR (p = 0.031) (Table 2). The cephalometric results also demonstrated that the median PAS improved significantly after robot-assisted TBR in the OSA subjects with huge lingual tonsils (postoperative – preoperative PAS: 12.5 mm (IQR: 9.8)). However, PAS was not significantly increased in the OSA patients with other TB abnormalities, although they underwent robot-assisted TBR (Table 3). Based on these results, we estimated that the therapeutic effect of robot-assisted TBR to widen retroglossal area would be relatively higher in OSA subjects with huge lingual tonsils following TBR.

**Table 2:** The comparison of sleep parameters and PAS between responders and non-responders following robot-assisted TBR.

	Responders (N = 10)	Non-responders (N = 6)	*p*-value
AHI	37.7/hr (IQR: 36.3)	60.2/hr (IQR: 49.6)	0.313
PAS	8.2 mm (IQR: 8.0)	18.0 mm (IQR: 4.6)	0.016
∆PAS	9.5 mm (IQR: 10.5)	3.5 (IQR: 4.8)	0.031

AHI: Apnea hypopnea index, PAS: Posterior airway space, ∆PAS: postoperative PAS - preoperative PAS.

**Table 3:** The change of PAS after robot-assisted TBR depending on TB abnormalities.

	Lingual Tonsil Hypertrophy	Other TB abnormalities	*p*-value
PAS	5.8 mm (IQR: 5.9)	12.9 mm (IQR: 4.3)	0.002
∆PAS	12.5 mm (IQR: 9.8)	4.0 mm (IQR: 4.3)	0.002

PAS: Posterior airway space, TB: Tongue base.

∆PAS: postoperative PAS - preoperative PAS.

## Discussion

Here, we found that robot-assisted TBR could be an adequate surgical option to improve upper airway narrowing at the retroglossal level in moderate to severe OSA. Our clinical findings also showed that there was a significant improvement of the upper airway width at the retroglossal level, indicating widened TB width after robot-assisted TBR and lingual tonsil hypertrophy might be the most favorable indication for robot-assisted TBR in OSA patients.

Impaired neuromuscular responses, failure to restore airway patency, and upper airway occlusion or narrowing aggravate physiologic airflow collapse during sleep. Excessive collapse of the upper airway increases the airway resistance and therefore contributes to the pathogenesis of OSA^21,22^. Therefore, widening or repositioning of the redundant tissues in the upper airway might be a critical therapeutic step for surgical treatment of OSA and it is essential to accurately identify severe collapse at each level of the upper airway to guide the appropriate surgical management of OSA patients^22^. Recently, DISE has been performed on most OSA subjects who underwent surgical treatment to investigate the level of upper airway collapse and the surgical options are considered based on the DISE findings^23^. DISE findings also provide the clinical information about the causes of TB narrowing during sleep such as lingual tonsil hypertrophy and our data suggest that favorable surgical indications for robot-assisted TBR can be decided based on DISE findings in OSA patients.

Compared to normal controls without OSA, patients with OSA tend to have smaller PAS at the level of retroglossal area indicating that TB narrowing might be one of the causes of development of OSA^24,25^. The TB is a deep-seated structure in the oropharynx; therefore, it is not only challenging to visualize this region during sleep surgery, but also to remove the hypertrophic base in OSA subjects^26^. According to several studies regarding the DISE findings in OSA patients, TB narrowing was a relatively common anatomic cause of aggravated intermittent hypoxia and oxygen desaturation in OSA patients. OSA patients with TB narrowing also exhibit higher AHI scores. However, it is difficult to effectively correct the TB narrowing in OSA patients^14,15,27^. Furthermore, the involved procedures are often associated with intra- or post-operative complications such as bleeding, respiratory difficulty, swallowing problems, dental injury, and taste change^26,28^. Given the anatomical location of TB, with its clinical significance in the pathogenesis of OSA, determination of surgical options for TBR would be critical to improve the therapeutic outcome of OSA surgeries.

Therefore, there is need for an adequate or safe surgical option to improve TB narrowing and to provide a satisfactory surgical outcome to OSA patients with severe retroglossal area collapse. The previous reports of open surgical approaches to TB, including two jaws surgeries, are now infrequently used given their higher morbidity, increased need for tracheostomy, and fatal complications. Other minimally invasive techniques are not useful in severe OSA patients^28^. In particular, transoral endoscopic techniques have <30% success with a relatively high risk of complications (5–25%)^12^.

Based on the previous literatures, robot-assisted TBR provides excellent three-dimensional images at the level of the TB and this technique allows for adequate correction of TB narrowing in OSA patients. In addition, robot-assisted TBR is practical and easy to use, which may improve the visualization with three-dimensional depth perceptions compared to that of traditional open surgeries^29,30^. The robotic technique seems to offer significant advantages in TBR and it enhances the ability of the surgeon in managing this complex region. With respect to traditional open approaches, robotic management of the TB seems to be very well tolerated^31^. In the present study, we have performed robot-assisted TB resection as part of multilevel surgery in moderate to severe OSA patients with severe TB narrowing and the decision to do robot-assisted TBR was based on DISE findings (DISE grade > II). Our clinical data demonstrate a significant decrease in AHI score and elevation of the lowest oxygen saturation in OSA patients with TB narrowing following robot-assisted TBR combined with nasal and palatal surgery. We also found that the extensive widening of regroglossal area was achieved through robot-assisted TBR and the clinical impact of robot-assisted TBR was more specifically in OSA patients with TB abnormalities such as lingual tonsil hypertrophy, TB narrowing despite of previous TB surgery, higher palatal grade.

To date, both endoscopic-guided coblation techniques and robot technology have been used (in lieu of open surgical approaches) to correct TB narrowing in OSA subjects^26,29,30^. Coblation techniques have provided better surgical outcomes to OSA subjects with TB narrowing accompanying with minimal complications and less morbidity^32–35^. However, robot-assisted TBR can be safely performed to correct TB narrowing without the need for tracheostomy or conversion to open surgery and the success rate of robot-assisted TBR in this study was comparable to that of endoscopic-guided TBR using coblation in the literature^29–31^. The current findings also showed that robot-assisted TBR as part of multilevel surgery, exhibited a success rate of approximately 62.5% in moderate or severe OSA. Patients who responded to surgery had no further CPAP requirements postoperatively and also experienced improved sleep quality with minimal discomfort. There was a low rate of complications and side effects related with robot-assisted TBR for up to three months after surgery. Tracheostomy was not necessary after robot-assisted TBR and the OSA patients required only two or more hospital days after robot-assisted TBR prior to discharge without staying in intensive care unit.

Actually, robot-assisted TBR exhibited a success rate less than 50% in OSA subjects with retrognathia and previous tongue base surgeries in accordance with a lesser increase in the PAS. Interestingly, robot-assisted TBR was more successful in OSA patients whose TB narrowing was due to huge lingual tonsils than in those without large tonsils and also produced a significant increase in the PAS and more complete AHI reduction. Therefore, we estimate that robot-assisted TBR provides good therapeutic outcomes in OSA patients with aggravated TB narrowing and is more effective (with regard to tongue base reduction and improving sleep parameters) in patients in whom the narrowing is caused by lingual tonsil hypertrophy.

Our study included patients with moderate or severe OSA with greater than 75% narrowing at the level of the tongue base and we expected such patients to require more potent volume reduction to improve TB narrowing. Endoscopic-guided TBR using a coblator has also obvious advantages in correcting TB narrowing but we believe that endoscopic-guided TBR provides inferior volume reduction (in TB widening) compared to that of robot-assisted TBR. Therefore, robot-assisted TBR creates a wider dimension in the upper airway at the level of the TB and reduces the bulk of the redundant soft tissue at the retroglossal area. Robot-assisted TBR can be performed safely with minimal injury to the lingual nerve, hypoglossal nerve, lingual artery, and taste buds and this is evidenced by the fewer complications of patients who underwent robot-assisted TBR than those who underwent TB surgery through open approach^29,30^.

The major current limitation of the present study was the surgical outcome and success rate of robot-assisted TBR was the result of combination with palatal and nasal surgeries, not single robot-assisted TBR. However, the presence of multilevel upper airway collapse has been implicated in OSA pathophysiology and it is hard to recruit the OSA subjects with only narrowing at the level of TB. In addition, the present study focused on searching the adequate indications of robot-assisted TBR and lingual tonsil hypertrophy appears to be most favorable surgical indications of robot-assisted TBR in patients with moderate or severe OSA. In summary, we sought to determine which OSA patients would benefit from robot-assisted TBR and to identify the favorable indications of robot-assisted TBR in the current study. We recommended robot-assisted TBR to OSA patients who are diagnosed with moderate or severe OSA with the following additional features: severe narrowing at the level of TB (DISE grade > II) due to huge lingual tonsils. Discussing the possible candidacy of robot-assisted TBR in OSA subjects is difficult and the number of included subjects in our series was relatively small. Nevertheless, our clinical findings present the clinical importance of robot-assisted TBR and are driven to avoid unnecessary robot surgeries in OSA subjects.
